# Evaluation of a Telemonitoring System Using Electronic National Early Warning Scores for Patients Receiving Medical Home Care: Pilot Implementation Study

**DOI:** 10.2196/63425

**Published:** 2024-12-26

**Authors:** Cheng-Fu Lin, Pei‐Jung Chang, Hui-Min Chang, Ching-Tsung Chen, Pi-Shan Hsu, Chieh-Liang Wu, Shih-Yi Lin

**Affiliations:** 1 Center for Geriatrics & Gerontology, Taichung Veterans General Hospital, 1650 Taiwan Boulevard Sect 4, Taichung, 407219, Taiwan, 886 4-2359-2525, 886 4-2359-5046; 2 Division of Occupational Medicine, Department of Emergency, Taichung Veterans General Hospital, Taichung, Taiwan; 3 Geriatrics and Gerontology Research Center, College of Medicine, National Chung Hsing University, Taichung, Taiwan; 4 Department of Post-Baccalaureate Medicine, College of Medicine, National Chung Hsing University, Taichung, Taiwan; 5 Home Health Care Agency, Taichung Veterans General Hospital, Taichung, Taiwan; 6 Department of Nursing, Taichung Veterans General Hospital, Taichung, Taiwan; 7 Computer & Communications Center, Taichung Veterans General Hospital, Taichung, Taiwan; 8 Department of Family Medicine, Taichung Veterans General Hospital, Taichung, Taiwan; 9 Department of Critical Care Medicine, Taichung Veterans General Hospital, Taichung, Taiwan; 10 Institute of Clinical Medicine, School of Medicine, National Yang Ming Chiao Tung University, Taipei, Taiwan; 11 Division of Endocrinology and Metabolism, Department of Internal Medicine, Taichung Veterans General Hospital, Taichung, Taiwan

**Keywords:** aging in place, early warning score, home hospitalization, remote monitoring, telemonitoring

## Abstract

**Background:**

Telehealth programs and wearable sensors that enable patients to monitor their vital signs have expanded due to the COVID-19 pandemic. The electronic National Early Warning Score (e-NEWS) system helps identify and respond to acute illness.

**Objective:**

This study aimed to implement and evaluate a comprehensive telehealth system to monitor vital signs using e-NEWS for patients receiving integrated home-based medical care (iHBMC). The goal was to improve the early detection of patient deterioration and enhance care delivery in home settings. The system was deployed to optimize remote monitoring in iHBMC and reduce emergency visits and hospitalizations.

**Methods:**

The study was conducted at a medical center and its affiliated home health agency in central Taiwan from November 1, 2022, to October 31, 2023. Patients eligible for iHBMC were enrolled, and sensor data from devices such as blood pressure monitors, thermometers, and pulse oximeters were transmitted to a cloud-based server for e-NEWS calculations at least twice per day over a 2-week period. Patients with e-NEWSs up to 4 received nursing or physician recommendations and interventions based on abnormal physiological data, with reassessment occurring after 2 hours.

**Implementation (Results):**

A total of 28 participants were enrolled, with a median age of 84.5 (IQR 79.3‐90.8) years, and 32% (n=9) were male. All participants had caregivers, with only 5 out of 28 (18%) able to make decisions independently. The system was implemented across one medical center and its affiliated home health agency. Of the 28 participants, 27 completed the study, while 1 exited early due to low blood pressure and shortness of breath. The median e-NEWS value was 4 (IQR 3‐6), with 397 abnormal readings recorded. Of the remaining 27 participants, 8 participants had earlier home visits due to abnormal readings, 6 required hypertension medication adjustments, and 9 received advice on oxygen supplementation. Overall, 24 out of 28 (86%) participants reported being satisfied with the system.

**Conclusions:**

This study demonstrated the feasibility of implementing a telehealth system integrated with e-NEWS in iHBMC settings, potentially aiding in the early detection of clinical deterioration. Although caregivers receive training and resources for their tasks, the system may increase their workload, which could lead to higher stress levels. The small sample size, short monitoring duration, and regional focus in central Taiwan may further limit the applicability of the findings to areas with differing countries, regions, and health care infrastructures. Further research is required to confirm its impact.

## Introduction

### Context

Home hospitalization, a patient-centered approach, offers benefits to individuals with chronic diseases by both reducing the frequency of their hospital visits and providing care within the comfort of their homes [1,2]. This type of model approach alleviates the burden of hospitalization, delivers more personalized care, and enhances medical efficiency [3,4].

### Problem Statement

In health care, connecting real-world objects with electronic devices such as wearable sensors for critical data collection has become essential. This integration is supported by cloud computing, which processes health data to better enhance human well-being. For instance, telemonitoring, widely adopted during the COVID-19 pandemic, uses pulse oximetry and symptom recording to detect any deterioration in one’s health [5]. Telemonitoring has become a transformative force in modern health care, offering numerous benefits while also addressing critical challenges [6]. The home monitoring of breathing rates in individuals diagnosed with chronic obstructive pulmonary disease holds promise, despite notable variability being reported in recovery rates [2]. Limited evidence suggests that potential benefits do exist for managing chronic diseases, including congestive heart failure and chronic obstructive pulmonary disease, when involving the use of telehealth programs and wearable sensors during medical emergency presentations. However, further research is still required in order to explore the effectiveness of patient-held monitoring devices across various health care settings, including after hospital discharge and during home contexts in the community [1].

### Similar Interventions

The early warning score is a comprehensive physiological scoring system that evaluates parameters such as respiratory rate, oxygen supplementation and saturation, body temperature, blood pressure, pulse rate, and level of consciousness. This scoring system has been formally recognized by the National Health Service in the United Kingdom and is used as an alert system to identify emergency patients in inpatient settings [7,8]. The value of the National Early Warning Score (NEWS) as a predictor of patient outcomes, particularly in the hospital, is that it can aid in longitudinal monitoring throughout each patient’s emergency department and hospital stays [8]. For example, the NEWS is recommended for the early detection and management of patients in the diagnosis and guidance of sepsis [9]. As for using the NEWS as an indicator of clinical changes in patients outside of inpatient settings, it is considered a valuable tool for aiding clinical decision-making [10]. Recent research indicates that using the NEWS in health care facilities can be a valuable supplementary tool for guiding emergency nursing decisions regarding residents in the nursing home, with there being a notable correlation between increasing NEWSs and longer hospital stays, as well as higher mortality rates [11]. Additionally, during the COVID-19 pandemic, identifying acute illnesses in nonhospital settings, particularly in care homes, became a global concern [12]. The British Geriatrics Society has recommended the adoption of the NEWS system to aid in this endeavor [10].

## Methods

### Aims and Objectives

The primary aim of this study was to establish and evaluate the feasibility of a comprehensive telemonitoring system integrated with the electronic NEWS (e-NEWS) system for monitoring vital signs in patients receiving integrated home-based medical care (iHBMC). Specifically, the study sought to assess the system’s ability to detect early signs of clinical deterioration and trigger timely interventions by health care professionals. Key objectives included evaluating the system’s completion rates for vital sign monitoring, identifying abnormal e-NEWS values and their associated interventions, measuring patient and caregiver satisfaction, and exploring operational challenges such as network connectivity and caregiver burden. This implementation report focused exclusively on the feasibility pilot study, aiming to highlight the lessons learned. The report was organized in accordance with the iCHECK-DH (Guidelines and Checklist for Reporting on Digital Health Implementations) [13].

### Blueprint Summary

**Figure 1.**
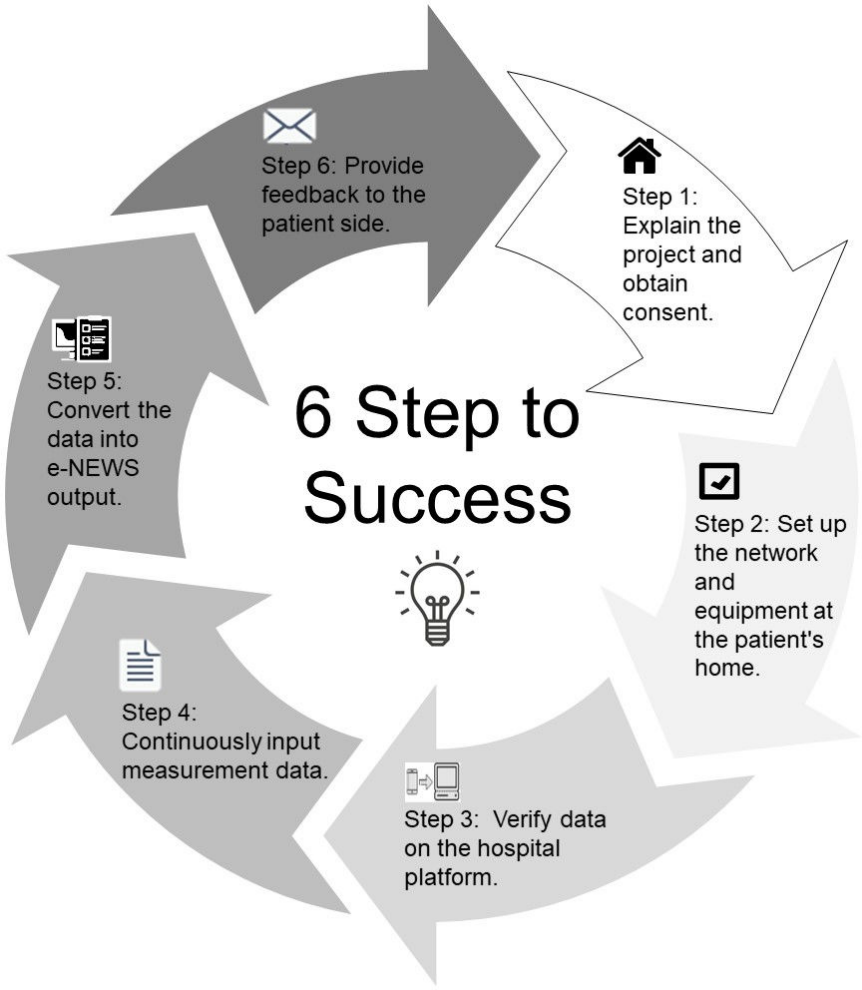
Development and implementation process of the e-NEWS–based mobile health care monitoring system. e-NEWS: electronic National Early Warning Score.

A multidisciplinary team comprising medical, technical, and support personnel was essential. The medical team included a physician responsible for overseeing patient care and clinical decision-making, a pharmacist tasked with conducting medication reviews and offering therapeutic recommendations, and a nurse case manager who monitored patients, responded to alerts, and coordinated care delivery. Furthermore, the team included a therapist and a nutritionist to address rehabilitative and nutritional needs, respectively. On the technical side, an information technology support engineer was required to maintain the telemonitoring systems, while a health informatics specialist managed the integration of telemonitoring data into the electronic health record (EHR). Additionally, the support staff included an administrative professional responsible for documentation management, billing, and maintenance of patient records, as detailed in Figure 1. This multidisciplinary approach ensured a coordinated, comprehensive framework for remote patient care.

### Technical Design

The QOCA (Quanta Omni Cloud Care; Quanta Computer Inc) APC (artificial intelligence patient care) system was selected due to its compatibility with Taiwan’s national health infrastructure, its integration capabilities with existing EHR, and its ease of use for patients and caregivers. The system supported various sensor devices, allowing for interoperability with different telemonitoring tools. The platform used secure data encryption to protect patient privacy, and the cloud-based nature of the system ensured scalability for potential nationwide implementation. The decision to use this system was also influenced by its adaptability for future upgrades, such as the integration of artificial intelligence to predict health deterioration.

### Target

Patients eligible for the iHBMC program, launched by the National Health Insurance (NHI) plan of Taiwan, resided in their own homes (excluding care facilities) and had a confirmed medical need as assessed by the health care team [14]. The medical need of each patient was attributed to either a disability or the nature of their illness, which rendered it challenging for them to access medical care outside their residences. The Barthel index assessed 10 domains of basic self-care tasks, including feeding, bathing, grooming, dressing, bowel and bladder control, toilet use, chair transfer, ambulation, and stair climbing. Each domain was scored based on the level of assistance required, with a total score ranging from 0 to 100, where higher scores indicated greater independence [11]. A score below 60 reflected significant dependence in performing daily activities, thereby qualifying as a measure of disability. The nature of one’s illness was assessed based on its severity, which included conditions such as severe dementia, leading to difficulties in seeking medical care outside of the home.

### Ethical Considerations

The research received approval from the institutional review board of the medical center (CE22459B), ensuring all procedures adhered to the established study protocol, standard regulations, and ethical principles outlined in the Declaration of Helsinki. Informed consent was obtained from all participants, who were informed of the study’s purpose, procedures, and their right to withdraw at any time. While the data were not anonymized, they were safeguarded through encryption, restricted access, and secure handling, with any issues managed by the medical center. In the event of adverse reactions or harm resulting from this study, compensation was provided by the medical center.

### Data

Patient demographics were investigated, including age, gender, disease diagnosis, medications, disease duration, comorbidities, and tube dependency, encompassing various types of medical tubes, such as the nasogastric tube, urinary catheter, and tracheostomy tube. Furthermore, the NEWSs comprised 7 vital signs: respiration rate, oxygen saturation, systolic blood pressure, heart rate, temperature, level of consciousness, and oxygen supplementation. Different scores were assigned based on varying degrees of abnormalities [7,8]. The e-NEWS is a modified version of NEWS, with the medium-risk threshold score reduced by 1 point to enhance patient safety [9]. For e-NEWS, a score of 0 to 3 implied continuation of the current treatment. If e-NEWSs fell between 4 and 6, patients were provided recommendations and interventions from a nurse or physician based on any abnormal physiological data, with their condition reassessed in 2 hours. If e-NEWSs were 7 or higher, it was strongly advised that the patient seek further medical evaluation, as confirmed by a multidisciplinary team. Additionally, if the patient’s vital signs did not deviate by more than one standard deviation, they were considered individual factors and were not subjected to intervention. If improvements could not be achieved through assessment, recommendations, or interventions, the visit schedule was adjusted to include an earlier home visit. All data were regularly updated at least twice per day for symptom recording and data collection over a 14-day period in accordance with recommendations from previous research [15,16]. Descriptive statistics were used to summarize and characterize the patient population and their health status, providing an overview of key demographic and clinical variables. This included measures of central tendency, dispersion, and frequency distributions. Furthermore, the graphical representation of data through charts and plots enhanced both the understanding and communication of findings. Statistical analyses were conducted with SPSS version 22.0 (IBM Corp).

### Interoperability

The telemonitoring system used standardized communication protocols, such as Fast Health Interoperability Resources, to ensure seamless data integration with existing hospital EHR systems. The use of these protocols allowed for real-time synchronization of patient data across different health care platforms, facilitating continuity of care between home-based care providers and hospital systems.

### Participating Entities

The telemonitoring system was implemented with collaboration from several key entities. The study was conducted at a medical center and its affiliated home health care agency in central Taiwan, where health care professionals including physicians, nurses, and pharmacists managed patient care and telemonitoring operations from November 1, 2022, to October 31, 2023. Technical support for the system was provided by Quanta Computer Inc, which developed the QOCA APC system, a cloud-based infrastructure for real-time monitoring of patient vital signs. The platform ensured smooth data transmission and timely alerts. Although the Taiwanese government did not directly fund the system, the iHBMC model supported by the NHI program aligned with national health care goals.

### Sustainability

The sustainability of the telemonitoring system depended on several factors, including the availability of funding, integration with existing health care infrastructure, and the long-term engagement of caregivers and patients. This study demonstrated the feasibility of the system within a short time frame; however, for widespread adoption, a sustainable business model was necessary. This included securing government support or public-private partnerships to offset the costs of system maintenance, training, and ongoing support for users. Future research also considered the environmental sustainability of telemonitoring systems, particularly regarding the use of electronic devices and data storage solutions.

## Implementation (Results)

### Coverage

**Figure 2.**
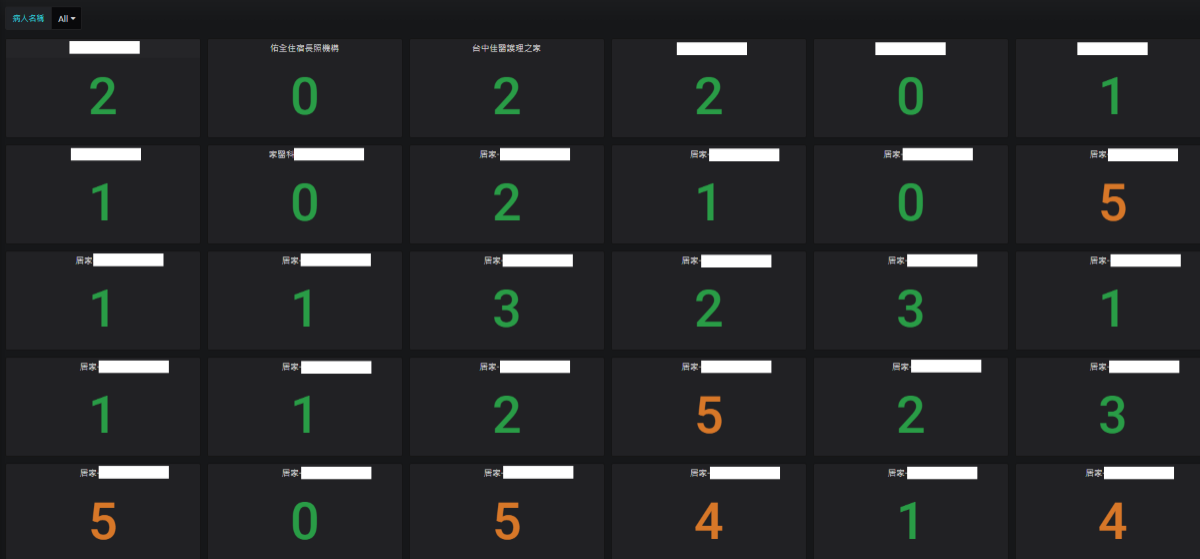
Architecture of the QOCA APC system and conversion of physiological data to e-NEWS format. APC: artificial intelligence patient care; e-NEWS: electronic National Early Warning Score; QOCA: Quanta Omni Cloud Care.

The telemonitoring system was implemented in central Taiwan, covering patients enrolled in iHBMC programs affiliated with a medical center. Although the study had a regional focus, the system had the potential for scalability to other regions across Taiwan. In this pilot phase, the study covered 28 patients, representing a small but significant portion of the eligible population within the hospital’s home care program. In Figure 2, the collected physiological data were converted into an e-NEWS format, providing a standardized indicator of patient conditions. These data were displayed on the cloud-based QOCA APC system, showing the status of virtual wards. When attention was needed, scores appeared in orange, allowing health care providers to click on them to investigate further, simplifying monitoring and inquiry.

### Clinical Outcomes

The baseline characteristics of the patients were depicted in Table 1, with the patients having a median age of 84.5 years and a predominance of females, accounting for 19 (68%) out of 28 patients of the cohort. The top 4 primary diseases among the 28 study patients, in order, were dementia with 11 patients (39%), diabetes mellitus with 10 patients (36%), cerebrovascular accident with 8 patients (29%), and congestive heart failure with 8 patients (29%). The patients demonstrated varying degrees of dependency in activities of daily living, with a median score of 0 (IQR 0‐18.8). Due to various disabilities, all patients lived with their family members and required care from others.

**Figure 3.**
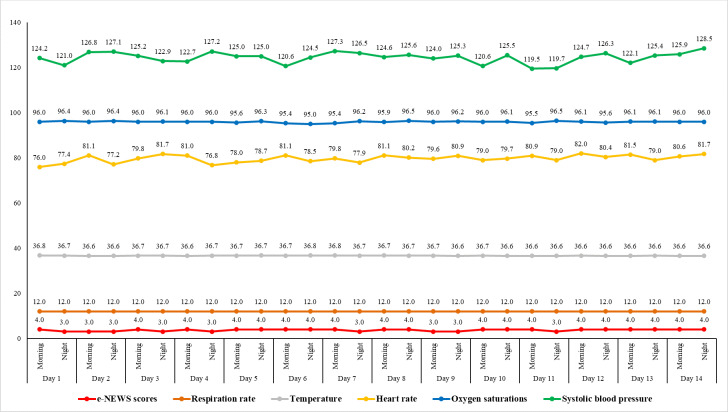
Patient vital signs and 14-day e-NEWS records. e-NEWS: electronic National Early Warning Score.

Tables2  3 present the percentage of e-NEWSs equal to or above 4, serving as an alert mechanism to notify health care providers of any score anomalies and to facilitate further interventions when necessary. After the vital signs were converted to e-NEWS, the overall median score was 4 (IQR 3‐6), with abnormal e-NEWS values recorded 397 times. Of these, 284 were classified as medium severity and managed by nurses, while 113 were categorized as high risk and handled in consultation with physicians. In Figure 3, patient records, including vital signs such as respiration rate, oxygen saturation, temperature, systolic blood pressure, and heart rate, were displayed alongside the 14-day e-NEWSs. Due to the automated alert system, the completion rate reached 100%, with only 1 patient out of 28 discontinuing due to hospitalization. The remaining 27 participants experienced no falls, emergency room visits, or hospitalizations. However, 8 patients had their home visits rescheduled earlier due to abnormal readings, 6 required medication adjustments for hypertension, and 9 received supportive advice for oxygen supplementation.

**Table 1.** Baseline characteristics of the participants (N=28).

Characteristics		Values
Age (years), median (IQR)		85 (79‐91)
**Gender, n (%)**		
	Female	19 (68)
	Male	9 (32)
BMI (kg/m^2^), median (IQR)		22 (20‐26)
**Educational level, n (%)**		
	Illiterate	7 (25)
	Primary school	13 (46)
	Junior high school	6 (21)
	Senior high school	1 (4)
	University	1 (4)
**Marital status, n (%)**		
	Single, widowed, or divorced	18 (64)
	Married	10 (36)
**Caregiver, n (%)**		
	Oneself	0 (0)
	Others	28 (100)
**Decision-making, n (%)**		
	Oneself	5 (18)
	Others	23 (82)
**Disease, n (%)**		
	Dementia	11 (39)
	Diabetes mellitus	10 (36)
	Congestive heart failure	8 (29)
	Cerebrovascular accident	8 (29)
	Solid tumor	7 (25)
	Myocardial infarction	5 (18)
	Peptic ulcer disease	4 (14)
	Chronic kidney disease	4 (14)
	Peripheral vascular disease	1 (4)
	Chronic obstructive pulmonary disease	1 (4)
	Connective tissue disease	1 (4)
Nasogastric tubes, n (%)		15 (54)
Urinary catheters, n (%)		14 (50)
Tracheostomy, n (%)		1 (4)
Age-adjusted Charlson comorbidity index, median (IQR)		7 (5‐9)
Polypharmacy, n (%)		16 (57)
Barthel index of activities of daily living, median (IQR)		0 (0‐19)
Glasgow coma scale, median (IQR)		12 (10-15)

**Table 2.** The percentage of vital signs above the medium level (n=27).

	Respiration rate, n (%)	Oxygen saturations, n (%)	Any supplemental oxygen, n (%)	Temperature, n (%)	Systolic blood pressure, n (%)	Heart rate, n (%)	e-NEWS^a^ value above medium level, n (%)
Day 1, morning	1 (4)	1 (4)	8 (30)	2 (7)	1 (4)	0 (0)	16 (59)
Day 1, night	1 (4)	2 (7)	7 (26)	2 (7)	0 (0)	0 (0)	12 (44)
Day 2, morning	1 (4)	3 (11)	7 (26)	2 (7)	3 (11)	0 (0)	12 (44)
Day 2, night	1 (4)	2 (7)	7 (26)	1 (4)	1 (4)	0 (0)	11 (41)
Day 3, morning	1 (4)	4 (15)	7 (26)	1 (4)	3 (11)	0 (0)	16 (59)
Day 3, night	1 (4)	1 (4)	7 (26)	0 (0)	4 (15)	0 (0)	12 (44)
Day 4, morning	1 (4)	2 (7)	7 (26)	1 (4)	5 (19)	0 (0)	15 (56)
Day 4, night	1 (4)	3 (11)	7 (26)	0 (0)	3 (11)	0 (0)	13 (48)
Day 5, morning	1 (4)	5 (19)	7 (26)	1 (4)	4 (15)	0 (0)	16 (59)
Day 5, night	1 (4)	3 (11)	7 (26)	1 (4)	3 (11)	0 (0)	14 (52)
Day 6, morning	1 (4)	6 (22)	7 (26)	1 (4)	4 (15)	0 (0)	16 (59)
Day 6, night	0 (0)	4 (15)	7 (26)	0 (0)	2 (7)	0 (0)	16 (59)
Day 7, morning	0 (0)	4 (15)	7 (26)	0 (0)	4 (15)	1 (4)	14 (52)
Day 7, night	1 (4)	2 (7)	7 (26)	0 (0)	2 (7)	0 (0)	13 (48)
Day 8, morning	1 (4)	3 (11)	7 (26)	0 (0)	3 (11)	0 (0)	15 (56)
Day 8, night	0 (0)	4 (15)	7 (26)	0 (0)	3 (11)	0 (0)	14 (52)
Day 9, morning	1 (4)	4 (15)	7 (26)	0 (0)	4 (15)	1 (4)	13 (48)
Day 9, night	1 (4)	2 (7)	7 (26)	0 (0)	3 (11)	0 (0)	13 (48)
Day 10, morning	2 (7)	4 (15)	7 (26)	0 (0)	4 (15)	0 (0)	14 (52)
Day 10, night	2 (7)	2 (7)	7 (26)	0 (0)	3 (11)	1 (4)	14 (52)
Day 11, morning	2 (7)	3 (11)	7 (26)	0 (0)	4 (15)	1 (4)	15 (56)
Day 11, night	2 (7)	2 (7)	7 (26)	0 (0)	3 (11)	0 (0)	12 (44)
Day 12, morning	2 (7)	4 (15)	7 (26)	0 (0)	4 (15)	0 (0)	14 (52)
Day 12, night	1 (4)	2 (7)	7 (26)	0 (0)	3 (11)	0 (0)	15 (56)
Day 13, morning	2 (7)	7 (26)	7 (26)	0 (0)	4 (15)	1 (4)	17 (63)
Day 13, night	1 (4)	1 (4)	7 (26)	0 (0)	4 (15)	0 (0)	15 (56)
Day 14, morning	2 (7)	5 (19)	7 (26)	0 (0)	7 (26)	0 (0)	16 (59)
Day 14, night	2 (7)	5 (19)	7 (26)	0 (0)	3 (11)	0 (0)	14 (52)

a e-NEWS: electronic National Early Warning Score.

**Table 3.** The percentage of vital signs above the high level (n=27).

	Respiration rate, n (%)	Oxygen saturations, n (%)	Any supplemental oxygen, n (%)	Temperature, n (%)	Systolic blood pressure, n (%)	Heart rate, n (%)	e-NEWS^a^ value above high level, n (%)
Day 1, morning	1 (4)	1 (4)	0 (0)	2 (7)	0 (0)	0 (0)	1 (4)
Day 1, night	1 (4)	1 (4)	0 (0)	2 (7)	0 (0)	0 (0)	3 (11)
Day 2, morning	1 (4)	1 (4)	0 (0)	2 (7)	0 (0)	0 (0)	5 (19)
Day 2, night	1 (4)	1 (4)	0 (0)	1 (4)	0 (0)	0 (0)	1 (4)
Day 3, morning	1 (4)	1 (4)	0 (0)	1 (4)	0 (0)	0 (0)	3 (11)
Day 3, night	1 (4)	1 (4)	0 (0)	0 (0)	1 (4)	0 (0)	2 (7)
Day 4, morning	1 (4)	2 (7)	0 (0)	1 (4)	1 (4)	0 (0)	4 (15)
Day 4, night	1 (4)	2 (7)	0 (0)	0 (0)	1 (4)	0 (0)	3 (11)
Day 5, morning	1 (4)	2 (7)	0 (0)	1 (4)	1 (4)	0 (0)	5 (19)
Day 5, night	1 (4)	1 (4)	0 (0)	1 (4)	2 (7)	0 (0)	2 (7)
Day 6, morning	1 (4)	1 (4)	0 (0)	1 (4)	1 (4)	0 (0)	5 (19)
Day 6, night	0 (0)	1 (4)	0 (0)	0 (0)	2 (7)	0 (0)	2 (7)
Day 7, morning	0 (0)	4 (15)	0 (0)	0 (0)	0 (0)	0 (0)	5 (19)
Day 7, night	1 (4)	1 (4)	0 (0)	0 (0)	1 (4)	0 (0)	2 (7)
Day 8, morning	1 (4)	2 (7)	0 (0)	0 (0)	1 (4)	0 (0)	4 (15)
Day 8, night	0 (0)	2 (7)	0 (0)	0 (0)	2 (7)	0 (0)	2 (7)
Day 9, morning	1 (4)	2 (7)	0 (0)	0 (0)	1 (4)	0 (0)	5 (19)
Day 9, night	1 (4)	1 (4)	0 (0)	0 (0)	2 (7)	0 (0)	4 (15)
Day 10, morning	2 (7)	2 (7)	0 (0)	0 (0)	1 (4)	0 (0)	6 (22)
Day 10, night	2 (7)	1 (4)	0 (0)	0 (0)	3 (11)	0 (0)	4 (15)
Day 11, morning	2 (7)	2 (7)	0 (0)	0 (0)	2 (7)	0 (0)	8 (30)
Day 11, night	2 (7)	1 (4)	0 (0)	0 (0)	3 (11)	0 (0)	4 (15)
Day 12, morning	2 (7)	2 (7)	0 (0)	0 (0)	3 (11)	0 (0)	8 (30)
Day 12, night	1 (4)	1 (4)	0 (0)	0 (0)	1 (4)	0 (0)	3 (11)
Day 13, morning	2 (7)	1 (4)	0 (0)	0 (0)	0 (0)	0 (0)	5 (19)
Day 13, night	1 (4)	1 (4)	0 (0)	0 (0)	1 (4)	0 (0)	4 (15)
Day 14, morning	2 (7)	2 (7)	0 (0)	0 (0)	1 (4)	0 (0)	7 (26)
Day 14, night	2 (7)	1 (4)	0 (0)	0 (0)	1 (4)	0 (0)	6 (22)

a e-NEWS: electronic National Early Warning Score.

### Patient Feedback and System Usability

Feedback regarding the use of the remote care system was collected and included information on user satisfaction, perceived effectiveness of the system, ease of use, and any technical issues or challenges encountered during its implementation (Table 4). Reasons for neutrality or dissatisfaction included 2 patients who were dissatisfied due to network connectivity issues and 2 others who felt they already had sufficient equipment for their care needs.

**Table 4.** Using feedback from e-NEWS^a^ (N=28).

		Patients, n (%)
**User experience**		
	Like	24 (86)
	Neutral	3 (11)
	Dislike	1 (4)
**Duration of use**		
	Full 14 days	27 (96)
	Less than 14 days	1 (4)

a e-NEWS: electronic National Early Warning Score.

### System Efficacy

The system achieved a 100% completion rate for vital sign monitoring, with only 1 patient withdrawing from the study due to hospitalization. The real-time alert system ensured prompt responses to abnormal vital sign readings, minimizing the risk of adverse events.

### Lessons Learned

Telemetric measurements using algorithms for risk assessment were still in the early stages [1,17,18], with internet stability being critical for prompt responses, similar to virtual wards [17,18]. In this study, we focused on the complex process of seamlessly connecting various devices to a single platform while simplifying data summarization and real-time monitoring. These features differed from those reported in other studies [18]. However, similar to other research, automated alerts to clinical staff for symptom changes led to 100% data collection success and ensured timely patient support [19]. The 14-day follow-up period maintained system stability and readiness, with no service disruptions.

Two key challenges in implementing an early warning score in primary care were the low probability of disease acuity and the weak correlation between NEWS and referral decisions. Understanding the full clinical impacts was crucial before the widespread adoption of the system in community settings [20]. Vital sign measurements were prone to inconsistencies due to numerous non–disease-related factors [21]. In older adults, particularly those who were frail, physiological deterioration did not always lead to rapid changes in vital signs [22]. While abnormal vital signs could predict emergency transfers from subacute to acute care hospitals, missed rapid response team activations during subacute care suggested the need for better recognition and response to patient deterioration [23]. Some studies on patients with advanced cancer showed that abnormal vital signs may signal impending death although some patients maintained normal readings until the end [21]. Proposed interventions for abnormal vital signs included lifestyle and medication advice, medication review and adjustments, video consultations, primary care referrals, home visits, secondary care referrals, and hospital admissions [24,25]. In our study, there were no falls or emergency visits among patients, but some home visits were rescheduled earlier due to abnormal readings. Comprehensive education and training for patients and caregivers on the proper use of medical devices and interpretation of NEWSs were critical for the system’s success. Health care providers offered workshops, web-based tutorials, and detailed guides to provide the necessary knowledge [26,27]. To support a nurse and caregivers, we provided training sessions, user guides, and technical support to ease the transition to e-NEWS monitoring and reduce burnout. Additionally, our patients, all of whom were in a state of disability, had dedicated caregivers, which may have been a key factor in the study’s success. Having consistent caregivers allowed for more accurate recognition of changes in patients’ conditions. The high level of caregiver involvement was a critical success factor in ensuring patient adherence to monitoring protocols and responding to alerts. However, challenges related to network connectivity affected the reliability of data transmission for some patients. Additionally, reliance on caregivers posed a potential barrier for patients without dedicated support, highlighting the need for additional resources in future implementations.

### Unintended Consequences

An unintended consequence of the study was the increased workload for caregivers, who had to manage the telemonitoring devices and respond to alerts. While this responsibility did not result in negative health outcomes, it may represent an additional burden for some families, particularly those with limited technical expertise.

## Discussion

### Summary of the Conclusions

Telehealth is being increasingly used in patient home care and may help address challenges while also supporting home-based patients who are receiving medical care by improving their independence, self-management, and access to community care services, as well as reducing unnecessary hospital admissions [28]. Adoption of the e-NEWS for use in home care settings represents a shift toward a more proactive and preventative approach to health care. By empowering both patients and caregivers to actively monitor a patient’s health status, the health care system can move away from reactive treatments and more toward preventing adverse health outcomes before they occur. Out of all our 28 patients, only 1 was unable to complete the 14 days of physiological monitoring, primarily due to multiple comorbidities and unstable vital signs. This patient was subsequently hospitalized and later passed away. The remaining 27 patients completed the monitoring successfully, with their feedback being mostly positive. Although some data alerts during monitoring required attention, this highlights the potential need for individualized care plans.

### Limitations

The small sample size of 28 participants and the short duration of monitoring limit our study, restricting the generalizability of the findings to broader populations. While the results provide valuable insights into the feasibility of e-NEWS in iHBMC settings, they may not fully apply to groups with diverse demographics, health care needs, or environmental conditions. Additionally, our study’s regional focus in central Taiwan may limit the applicability of findings to regions with different health care infrastructures, caregiver resources, and technology access. Studies involving larger and more diverse patient populations across varied geographic settings would offer stronger evidence for broader application.

### Future Implications

In this pilot study, e-NEWSs serve as a critical part of clinical decision-making, helping identify patients with abnormal physiological readings and prompting timely interventions. Patients with medium to high e-NEWSs trigger specific actions by the multidisciplinary team, such as medication adjustments, supplemental oxygen, and rescheduling of home visits for closer monitoring. This scoring system appears effective in preventing clinical deterioration, as suggested by a reduction in emergency room visits and a high completion rate of health assessments. However, further research with a broader cohort is essential to determine whether these trends consistently lead to positive outcomes and reduced hospital use across varied care environments.

The implementation also affects caregivers, who are responsible for monitoring devices, interpreting alerts, and supporting patient care based on e-NEWS indicators. Although caregivers receive training and resources to handle these responsibilities, the system adds to their daily workload, potentially increasing caregiver strain. To mitigate this, strategies such as ongoing technical support, streamlined device functionality, and educational workshops are used to ensure caregivers can manage these tasks without compromising their well-being. Future iterations of the e-NEWS system could further enhance caregiver support, incorporating features that reduce manual input and provide automated guidance, enabling more efficient caregiving while maintaining system effectiveness.

## Supplementary material

10.2196/63425 Checklist 1 iCHECK-DH

## Abbreviations

APC: artificial intelligence patient care
EHR: electronic health record
e-NEWS: electronic National Early Warning Score
iCHECK-DH: Guidelines and Checklist for Reporting on Digital Health Implementations
iHBMC: integrated home-based medical care
NEWS: National Early Warning Score
NHI: National Health Insurance
QOCA: Quanta Omni Cloud Care
